# Genetic Diversity and Evolution of the Biological Features of the Pandemic SARS-CoV-2

**DOI:** 10.32607/actanaturae.11337

**Published:** 2021

**Authors:** A. A. Nikonova, E. B. Faizuloev, A. V. Gracheva, I. Yu. Isakov, V. V. Zverev

**Affiliations:** Mechnikov Research Institute for Vaccines and Sera, Moscow, 105064 Russia

**Keywords:** coronaviruses, SARS-CoV-2, pathogenicity, virulence, contagiousness, virus evolution, viral genome

## Abstract

The new coronavirus infection (COVID-19) represents a challenge for global health. Since the outbreak began, the number of confirmed cases has exceeded 117 million, with more than 2.6 million deaths worldwide. With public health measures aimed at containing the spread of the disease, several countries have faced a crisis in the availability of intensive care units. Currently, a large-scale effort is underway to identify the nucleotide sequences of the SARS-CoV-2 coronavirus that is an etiological agent of COVID-19. Global sequencing of thousands of viral genomes has revealed many common genetic variants, which enables the monitoring of the evolution of SARS-CoV-2 and the tracking of its spread over time. Understanding the current evolution of SARS-CoV-2 is necessary not only for a retrospective analysis of the new coronavirus infection spread, but also for the development of approaches to the therapy and prophylaxis of COVID-19. In this review, we have focused on the general characteristics of SARS-CoV-2 and COVID-19. Also, we have analyzed available publications on the genetic diversity of the virus and the relationship between the diversity and the biological properties of SARS-CoV-2, such as virulence and contagiousness.

## INTRODUCTION

After the first cases of infection were reported in Wuhan, China, in December 2019, the novel coronavirus infection COVID-19 caused by SARS-CoV-2 spread throughout the world and became the first coronavirus pandemic in human history [1]. As of March 2021, COVID-19 had been diagnosed in more than 117 million people worldwide, with more than 2.6 million deaths [2]. Currently, preventive vaccines are far from available in all countries or to all segments of their populations; therefore, quarantine, social distancing, and special sanitary precautions have remained the most potent measures to prevent the further spread of the infection.

The rapid and worldwide spread of the new coronavirus is inevitably associated with its divergence and the emergence of strains with various biological properties, the most significant of which is virulence. Very little is known about the phenotypic diversity of SARS-CoV-2, given the short period during which it has been investigated. Unfortunately, the available reports on genomic sequences provide limited information about the patient and are confined to age and gender, while, often, information on the severity, manifestations, and outcome of the disease is lacking.

One of the topical issues of fundamental and medical virology is the identification of the nature of the pathogenicity and virulence of viruses, including those of animal origin. Despite the progress made in understanding the evolution of viruses, the question of the evolution of virulence resulting from interspecies transmission remains open. Does the virus become more or less virulent in a new host? How is the degree of virulence modulated by natural selection and why? Are there regularities in the evolution of virus virulence in a new host which can allow one to predict the direction of this process? A simplified interpretation of virulence evolution is that natural selection optimizes the level of virulence in a way that increases the efficiency of viral transmission, which is characterized by the basic reproduction number (R0) [3]. The adaptation of a virus to a new host is affected by a complex set of host–pathogen interactions. According to modern concepts, during interspecies transmission, the initial virulence of a virus can vary from an absence of pathogenicity (asymptomatic carriage) to a high pathogenicity, while it remains very difficult to predict the direction in which virulence will evolve. Mankind has rarely faced a highly virulent pandemic virus of animal origin – once every several decades – but the consequences of such an occurrence are dire and often global in scope. In such a context, it is of extreme importance to understand and predict how the biological properties of the SARS-CoV-2 coronavirus can evolve. The purpose of this review is to analyze the results of scientific studies that have focused on the relationship between genetic changes in the SARS-CoV-2 virus and its biological properties, including pathogenicity, virulence, and contagiousness.

The *pathogenicity *of a virus is defined as its ability to cause a disease. The term “virulence” can have different meanings depending on the context. In this review, the *virulence *of a virus means the measure of its pathogenicity; i.e., its ability to cause more or less severe diseases; the degree of virulence is determined by the mortality rate. The *contagiousness *(transmissibility) of a virus is its ability to move from infected organisms to healthy ones. Contagiousness is assessed with two interrelated indicators: the contagiousness index (the proportion of susceptible persons infected after contact with a source of the pathogen) and the basic reproduction number R0 (the average number of cases directly infected by one case during the entire infectious period in a completely susceptible population).

## GENERAL CHARACTERIZATION OF SARS-CoV-2

The pandemic SARS-CoV-2, along with the SARS-CoV virus, belongs to the Coronaviridae family, Orthocoronavirinae subfamily, *Betacoronavirus *genus,* Sarbecovirus *subgenus, and *Severe acute respiratory syndrome-related coronavirus *species [4]. It should be noted that, along with the listed pathogens, the *Sarbecovirus* subgenus also includes coronaviruses isolated from bats; in particular, horseshoe bats (*Rhinolophus* genus) [5]. The genome sequence of SARS-CoV-2 was found to be 96.2 and 93.3% identical to that of the raTG13 [6] and RmYN02 [7] bat coronaviruses, respectively. The degree of nucleotide sequence similarity and evolutionary analysis lends credibility to the hypothesis that bats are the natural reservoir of the SARS-CoV-2 that was transmitted to humans through unknown intermediate hosts [8, 9]. In addition, the SARS-CoV-2 genome has been shown to be 85.5–92.4% similar to that of coronaviruses isolated from pangolins [10], 80% to SARS-CoV [6], and 50% to MERS-CoV (*Merbecovirus *subgenus) [11]. However, the degree of genome homology varies greatly depending on genes and genomic loci [5]. In this case, the main differences between these viruses reside in the *ORF1a *sequence and the gene encoding the spike protein S that plays a key role in the interaction of the virus with the cell [12]. These features of genome organization may be a result of some interviral recombination [13].

SARS-CoV-2 virions are pleiomorphic (usually spherical), with an average diameter of 108 ± 8 nm, ranging from 84 to 126 nm [14]. The spikes on the surface of viral particles, about 9–12 nm long, give the virus its characteristic crown appearance. The morphology of SARS-CoV-2 virions is similar to that of other members of the Coronaviridae family, including SARS-CoV and MERS-CoV [15].

**Fig. 1:**
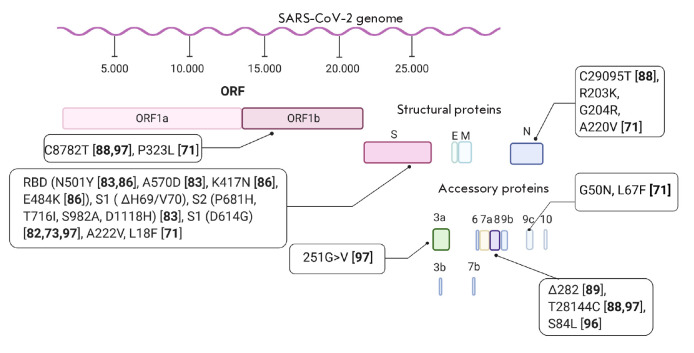
Major mutations in the SARS-CoV-2 genome which were identified within a year of the beginning of the pandemic (created with the online software BioRender)

The SARS-CoV-2 genome is a nonsegmented, single- stranded, positive sense RNA, 29.9 kb in size, and consists of six main open reading frames (ORF) (*Fig. 1*). Translation of virus-encoded RNA-dependent RNA polymerase (replicase) is necessary for the initiation of viral replication in the cell and the synthesis of the subgenomic viral RNAs that, in turn, serve as a matrix for the synthesis of viral structural and accessory proteins [16]. The size of *ORF1ab*, which encodes replicase, is 2/3 of the size of the entire viral genome. *ORF1ab *is followed by the genes for the spike protein (*S*), *ORF3a*, envelope protein (*E*), membrane protein (*M*), *ORF6*,* ORF7a*, *ORF7b*, *ORF8*, nucleocapsid (*N*), and *ORF10*. In addition, Nelson *et al*. proved that SARS-CoV-2 contains a new overlapping gene (OLG) *ORF3d *[17] that is also present in coronaviruses isolated from pangolins in the Guangxi region of Southern China, but that it is not found in other coronaviruses isolated from pangolins and bats.

**Fig. 2:**
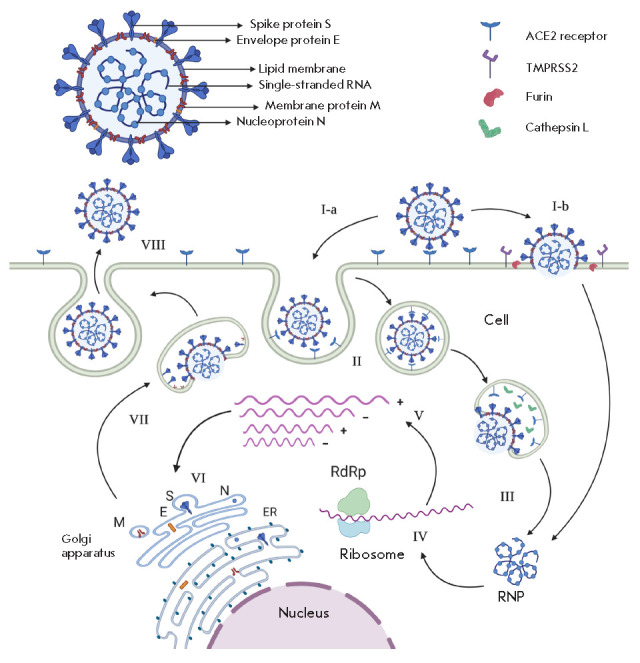
The SARS-CoV-2 virion and life cycle (created with the online software BioRender) I. Virus binding. After adsorption, the virus can enter the cell in two ways: through the endosome (I-a) or through fusion with the cell membrane (I-b) II. Receptor-mediated endocytosis III. Fusion of the virus envelope with the endosome membrane results in virus uncoating. Release of the ribonucleoprotein complex (RNP) IV. Viral genome translation. Synthesis of the viral proteins (including RNA-dependent RNA polymerase (RdRp) involved in genome replication and transcription V. Replication and transcription of the viral genome VI. Viral proteins are synthesized at the endoplasmic reticulum (ER) lumen VII. Assembly and transport of the virions to the cell membrane VIII. Release of the virions by exocytosis

The spike protein S of the SARS-CoV and SARS-CoV-2 coronaviruses initiates a fusion of the viral envelope with the cell membrane of the host cell, and the angiotensin-converting enzyme 2 (ACE2) serves as a cellular receptor for the attachment of the virus. The receptor for MERS-CoV is hDPP4 (human dipeptidyl peptidase 4 or CD26) [18]. The S protein comprises two domains, S1 and S2. The S1 domain mediates the binding to ACE2, while the S2 domain mediates subsequent fusion of the viral envelope with a cell’s membrane [19]. The receptor binding domain (RBD) is a key functional component of S1, which is responsible for the binding of SARS-CoV-2 to ACE2 [20]. In addition, the SARS-CoV RBD contains a core motif and a receptor binding motif (RBM) that mediates the contacts with ACE2. The surface of ACE2 contains two virus-binding hotspots that are essential for SARS-CoV-2 binding [21]. The stage of adsorption and penetration of SARS-CoV-2 into a cell depends not only on the ACE2 receptor, but also on the transmembrane serine protease TMPRSS2 and proprotein convertase furin, whose role is to prime the SARS-CoV-2 S protein [22, 23]. Thus, SARS-CoV-2 can enter a cell in two different ways (*Fig. 2*): through the late endosome where the S protein is cleaved by cathepsins, or through the cell membrane or early endosome using trypsin-like proteases to cleave the S protein [23, 24].

The E protein forms ion channels and regulates the assembly of virions [25]. The M protein is also involved in the assembly of viral particles [26], while the N protein forms a ribonucleoprotein complex with viral RNA and performs several functions, such as enhancing the transcription of the viral genome and interacting with the viral membrane protein during virion assembly [27].

The receptor of target cells, which is used by the virus to enter a cell, is a factor in determining which organs and tissues are susceptible to infection. The ACE2 receptor is expressed on the surface of epithelial cells of the alveoli, trachea, bronchi, and bronchial glands, as well as on alveolar macrophages. In addition, ACE2 is present on mucous membranes, such as the cornea of the eye and goblet and ciliary cells in the nasal cavity [28], which appear to be the gateway to infection. The life cycle of the virus with the host consists of the following steps: the virus enters the cell using the ACE2 receptor and releases the single-stranded viral RNA that binds to the target cell’s ribosome and initiates the synthesis of the RNA replicase that, in turn, reproduces copies of genomic and subgenomic RNA, as well as RNA fragments that serve as templates for the synthesis of viral envelope proteins. Positive-sense viral RNA molecules, together with structural viral proteins, form new SARS-CoV-2 virions that are released from the cell and infect intact target cells (*Fig. 2*) [29].

## VARIETY OF CLINICAL MANIFESTATIONS OF COVID-19

The severity of the disease caused by SARS-CoV-2 can vary significantly [30]. There is great variability in the clinical presentations of COVID-19 even among close contacts of an infected person or members of the same family [30]. The spectrum of COVID-19 symptoms ranges from mild/moderate to critical and fatal [31, 32, 33]. Also, an asymptomatic course of the disease is often observed. The rate of asymptomatic cases can amount to 40–50%, and an infected person remains a source of infection for more than 14 days [34]. In addition, an asymptomatic course of the infection can be associated with subclinical changes in the lungs, which are detected during computed tomography [34]. Therefore, SARS-CoV-2 possesses increased virulence with a tactical advantage – the ability to maintain human-to-human transmission even in asymptomatic carriers [35], which allows the virus to spread rapidly.

According to a report issued by the Chinese Center for Disease Control and Prevention [36], an analysis of 44,500 confirmed cases of infection with an assessment of the disease severity revealed that a mild form of COVID-19 (nonpneumonia and mild pneumonia) is observed in 81% of cases. A severe form (dyspnea, hypoxia, or lung involvement of >50%) was reported in 14% of cases. And 5% of cases were critical (respiratory failure, shock, or multiple organ dysfunction). In this case, the overall mortality rate was 2.3% (no deaths among non-critical cases).

A severe form of the COVID-19 disease can be observed in any healthy person of any age, but it occurs mainly in people over 65 years of age and/or with concomitant diseases (cardiovascular diseases, diabetes mellitus, hypertension, chronic lung and kidney diseases, cancer, obesity, smoking) [32, 36, 37], while, in most young adults, the infection is mild and without complications.

There are several complications associated with COVID-19. These include the acute respiratory distress syndrome, which is a type of respiratory failure that requires critical care support, including artificial ventilation of the lungs. This care is required in 12 to 24% of hospitalized patients [38, 39]. Also, cardiovascular [40] and thromboembolic complications [41], inflammatory reactions [42], and superinfections [43] are observed.

Children are the least susceptible to infection. They account for 1 to 6.3% of COVID-19 cases [44, 45]. According to a report by China’s Center for Disease Control and Prevention, of the 72,314 cases reported as of February 11, 2020, only 2% were under the age of 19 [36]. Multisystem inflammatory syndrome with clinical signs similar to Kawasaki’s disease and toxic shock syndrome were reported in children with COVID-19 [46]. Monitoring of children infection by Meskina [45] showed that the rate of asymptomatic COVID-19 cases in children was 62%, with that in newborns being 73.1%, and the rate of severe forms being as low as 0.38%.

## GENETIC DETERMINANTS OF SARS-CoV-2 VIRULENCE

The question of the causes behind the diverse clinical presentations of COVID-19 in different categories of the population remains open. It may be that this diversity depends on certain genetic profiles of a host organism. In accordance with this hypothesis, the genetic basis of the susceptibility to infection may be explained by the polymorphism of the functional receptors required for virus entry into target cells. In particular, multiple organ dysfunctions in COVID-19, including fatal damage to the lungs and myocardium, may be associated with the functional characteristics of the ACE2 receptors in the population [47, 48, 49]. For example, Hou *et al.*, based on an analysis of ~ 81,000 human genomes, investigated the association between the polymorphism of the *ACE2 *and *TMPRSS2 *genes (two key host factors of SARS-CoV-2) and susceptibility to COVID-19. *ACE2 *polymorphisms (p.) (e.g., p.Arg514Gly in the African/African American population) were found to be associated with the cardiovascular and pulmonary diseases through altered angiotensinogen– ACE2 interactions. Unique but prevalent polymorphisms (including p.Val160Met (rs12329760)) in *TMPRSS2 *have the potential to cause differential genetic susceptibility to COVID-19 [50].

A genome-wide association study (GWAS) analyzed 8,582,968 single nucleotide polymorphisms (SNPs) from 1,980 severe COVID-19 patients from the Italian and Spanish epicenters of the pandemic in Europe. The study did not reveal any significant associations of a severe form of the disease with a single gene. Rather, it did so with a multigene cluster on chromosome 3 (*SLC6A20*, *LZTFL1*, *CCR9*, *FYCO1*, *CXCR6*, and *XCR1* genes) [51].

Chinese scientists analyzed the genetic profiles of 332 patients with varying severity of COVID-19 using NGS techniques. The results of a genome-wide association study (GWAS) indicated that the most significant locus associated with disease severity was located in *TMEM189*–*UBE2V1, *which is involved in the interleukin-1 (IL-1) signaling pathway. The rate of p.Val197Met missense variants of the *TMPRSS2* gene, which affect the stability of the protein, is lower in patients with a severe infection than in patients with a mild form of the disease and the general population. In addition, the HLA-A*11:01, B*51:01, and C*14:02 alleles were found to significantly predispose people to a severe course of COVID-19 [52].

Selectivity for host genetic profiles (as a factor of SARS-CoV-2 virulence) may be one of the viral features. This property was not reported in the influenza virus which caused the global pandemic in 1918. This may be because, in the early 1900s, the level of technological evolution and knowledge did not allow for conducting research on the topic. There is data indicating that susceptibility to HIV-1 is genetically determined by variations in the host chemokine receptors [53]. This data suggests that this selectivity may determine the virulence and tissue specificity of other viruses, including SARS-CoV-2.

Studies of the molecular mechanisms of the pathogenicity and contagiousness of coronaviruses have focused on the determinants of coronavirus tropism to the cells of the human respiratory tract, which is associated with receptor-mediated virus entry into the cell. These determinants are present on the surface S protein of coronaviruses. Mutations in S protein epitopes, which are responsible for the binding to viral receptors, are believed to determine the efficiency of interspecies transmission and adaptation of the virus to a new host [54]. There is experimental evidence that the bat coronavirus, whose S-protein can be modified by reverse genetics methods, is able to overcome the species barrier (to infect human cells) [55]. However, to date, there has been no experimental confirmation that the SARS-CoV-2 S protein alone mediates contagiousness or the high virulence of the virus in humans. Previously, a highly pathogenic avian influenza A virus, H5N1, was used to prove that an ability to recognize the viral receptors of epithelial cells in the respiratory tract of mammals could be achieved by introducing two to four amino acid substitutions into hemagglutinin (HA), which are essential for the binding of HA to α-2,6-sialic receptors [56, 57]. However, these mutations alone were not enough for a virus to acquire contagiousness and high virulence for ferrets [56, 57]. This indicates that additional determinants of contagiousness and virulence are likely encoded in the internal genes of the virus. The pathogenicity of the virus is mediated not only by its ability to effectively penetrate target cells, but also by many other viral factors. An example of this is vaccine strains that are used as live, attenuated vaccines. According to Klimov *et al. *[58], the determinants of the attenuation of the cold-adapted vaccine strain of influenza A/Leningrad/134/47/17 are mutations in the genes of the polymerase complex proteins (PB1, PB2, PA, NP), M-protein, and the nonstructural protein NS2, but not in the genes of the surface proteins neuraminidase N and hemagglutinin H. Review [3] provides more than 10 examples of changes in the virulence of various mammalian viruses which are caused by only one or two amino acid substitutions. Most of these examples concern RNA viruses (influenza A and B viruses, enteroviruses, Ebola virus, HIV, West Nile virus, Newcastle disease virus, porcine reproductive and respiratory syndrome virus, etc.).

Viruses of the same biological species can significantly differ in virulence, something associated with divergence in the course of evolution. The mortality rate from an infection with seasonal influenza A viruses (*Influenza A virus *species of the Orthomyxoviridae family) of the H3N2 and H1N1 serotypes is 0.04–1.0%, while that from diseases caused by some strains of the avian influenza A virus, including H5N1, H7N7, H9N2, H7N3, and H7N9, reaches 60% [59, 60]. Human coronaviruses are no exception. The so-called seasonal coronaviruses (HCoV-NL63, -229E, -OC43, -HKU1) are associated mainly with mild and moderate forms of acute respiratory viral infections, while coronaviruses of animal origin (SARS-CoV, MERS-CoV, and SARS-CoV-2) are associated with the development of a severe acute respiratory syndrome and a higher risk of mortality (according to various estimates, 1 to 40% of the number of laboratory-confirmed cases).

Recently, the Koonin’s group identified possible genetic determinants for the increased mortality from an infection with the highly virulent coronaviruses SARS-CoV, MERS-CoV, and SARS-CoV-2 compared to that from the low-virulent seasonal HCoV-NL63, -229E, -OC43, and -HKU1 [61]. An analysis of more than 3,000 coronavirus genomes revealed that the genome of the highly pathogenic coronaviruses SARS-CoV, MERS-CoV, and SARS-CoV-2 contains four regions (three in the N nucleoprotein gene and one in the S protein gene) significantly different in amino acid sequences from seasonal coronaviruses [61]. The differences in the *N *gene presumably determine the enhancement of signals for nuclear localization and export of this protein. Differences in the *S *gene occur at the site of receptor recognition and fusion of the viral envelope with the cell membrane and are hypothetically responsible for enhancing the stage of virus attachment and entry into the cell. The obtained results shed light on the potential determinants of coronavirus virulence, but they have not yet been empirically confirmed, because the work was performed using computer-based analysis methods.

New mutations constantly occur in the genome of any virus, and some of these are capable of changing the biological properties of the virus, including the degree of contagiousness, ability to evade the host’s immune response, and virulence. The viral RNA genome of SARS-CoV-2 is characterized by a high mutation rate (but lower than that of other RNA viruses) [62].

To date, hundreds of thousands of genome sequences for the SARS-CoV-2 coronavirus are known. The results of multiple studies have enriched the GISAID genome sequence database, which, as of January 2021, includes information on more than 323,493 sequences. In addition to SARS-CoV-2, GISAID contains the genome sequences of coronaviruses isolated from bats and pangolins. Based on data from viral sequences and information on the geographical origin of the samples in GISAID, another information resource, Nextstrain (https://nextstrain.org) [63] publishes hosts phylogenetic, geographic, and genomic analyses of SARSCoV- 2. Using the GISAID database and Nextstrain resource, the evolution of a virus can be monitored in real time. The Nextstrain analysis predicts the occurrence of approximately 26 substitutions in the SARS-CoV-2 genome per year. Given the SARS-CoV-2 genome size (29.9 kb), the estimated evolutionary rate is approximately 0.90 × 10–3 substitutions/site/year [5]. This value is comparable with values reported for SARS-CoV (0.80–2.38 × 10^–3^) [64], MERS-CoV (0.63–1.12 × 10^–3^) [65], and HCoV-OC43 (0.43 × 10^–3^) [66]. Since the coronavirus genome encodes a 3’–’-exoribonuclease (nsp14-ExoN) that has editing activity, the mutation rate (the number of single nucleotide substitutions per site per replication cycle) is likely to be lower in SARS-CoV-2 than in other RNA viruses, such as influenza viruses [67]. This underlies the high stability of the genome of coronaviruses, including SARS-CoV-2. An analysis of the nucleotide sequences of 48,635 virus isolates confirmed the low mutation rate, which was 7.23 mutations per sample, on average, compared with the reference SARS-CoV-2 genome (NC_0455122) [68].

In addition, the SARS-CoV-2 genome was shown to have a much lower mutation rate and genetic diversity compared to those of the SARS-CoV virus that caused the outbreak of SARS in 2002–2003 [69]. It should also be noted that the S protein RBD domain (~90 aminoacids) of SARS-CoV-2, which reacts directly with the ACE2 receptor on the surface of target cells, differs significantly from the SARS-CoV RBD, especially in two regions that interact with ACE2, and is the part of SARS-CoV-2 most susceptible to variations [70]. The latter suggests the participation of several mechanisms involved in virus entry into the cell. Six amino acid residues of the S protein RBD (Leu455, Phe486, Gln493, Ser494, Asn501, and Tyr505) were found to play a key role in the binding to ACE2. In this case, five of them differ from the SARS-CoV RBD, which should be considered in the development of specific antiviral agents that block virus entry into the cell [70].

It should be noted that numerous elements of the virus genome are mutated at different rates. For example, an analysis of about 223,000 full-length sequences of the SARS-CoV-2 proteome was used to calculate the mutation rate of each viral protein. The highest mutation rates were observed in the S, NSP12, NS9c, and N proteins [71].

An analysis of the nucleotide sequences of SARS-CoV-2 isolates revealed several genome regions with an increased mutation rate [72, 73, 74, 75, 76, 77, 78, 79, 80, 81]. One of these regions is D614G, in the C-terminal region of the S1 domain [72, 73, 74, 77, 81]. A mutant virus with a D614G substitution in the S1 domain was shown to be prevalent in Europe [68]. Comparison of the functional properties of the S protein with aspartic acid at position 614 (SD614) and glycine (SG614) showed that pseudoviruses carrying SG614 enter ACE2-expressing cells more efficiently than viruses with SD614 [82]. While evidence continues to accumulate, a growing proportion of the virus with the D614G substitution suggests that viruses with this mutation are characterized by a more efficient personto- person transmission. Interestingly, this mutation does not appear to significantly affect the severity of the disease [73, 79].

In December 2019, isolation of a new SARS-CoV-2 strain with an increased level of contagiousness was reported in the UK [83]. According to the data of a phylogenetic analysis, this strain forms a distinct phylogenetic cluster (lineage B.1.1.7) [84]. Seven characteristic mutations were identified in the S protein of this virus: RBD (N501Y, A570D), S1 (ΔH69/V70), and S2 (P681H, T716I, S982A, and D1118H) [83]. The N501Y mutation in the receptor binding domain (RBD) provides increased affinity for human and mouse ACE2 [85]. The ΔH69/V70 deletion in S1 enhances the ability of the virus to evade the immune response. The P681H mutation is directly adjacent to the furin cleavage site between S1 and S2 in the S protein. In addition, there is data pointing to the existence of several independent SARS-CoV-2 lineages that are characterized by the ΔH69/V70 deletion in the S protein and an increase in the circulation of these viruses in some European countries since August 2020 [83].

In January 2021, a new SARS-CoV-2 (501Y.V2) lineage emerged in South Africa. It quickly spread and became prevalent in several regions of the country. There are eight S protein mutations characteristic of this lineage; in particular three in the RBD (K417N, E484K, and N501Y) which may be of functional value. Two of these (E484K and N501Y) are located in the receptor binding motif (RBM) that directly interacts with ACE2 [86]. The N501Y mutation is also characteristic of the B.1.1.7 lineage identified in the UK. Perhaps, this mutation determines the level of SARS-CoV-2 contagiousness.

There are also reports of a new SARS-CoV-2 P.1. lineage in Brazil [87]. It is necessary to note the emergence of convergent mutations common to the P1, B.1.1.7, and B.1.351 lineages (*Table 1*). These are the N501Y mutation in the S protein and a deletion in ORF1b (del11288–11296 (3675–3677 SGF)) common to P.1. and the British B.1.1.7, as well as mutations in the RBD (K417N/T, E484K, N501Y) typical of both P.1. and the South African B.1.351.

**Table 1:** Major genetic variants of SARS-CoV-2

Genetic SARS-CoV-2 variant	Region where it was first detected	Typical mutations	Characteristic features
B.1.1.7	United Kingdom	S protein: RBD (N501Y, A570D), S1 (ΔH69/V70) S2 (P681H, T716I, S982A, and D1118H)	High contagiousness
B.1.351 (N501Y.V2)	Republic of South Africa	S protein: RBD (K417N, E484K, and N501Y)	Some vaccines are less effective against this variant, high contagiousness
P1 descendant of B.1.1.28	Brazil	S protein: RBD (E484K, K417T, and N501Y)	High contagiousness
Fin-796H	Finland	S protein: RBD (E484K, K417T, and N501Y)	Not detectable in PCR

The set of mutations/deletions characteristic of the P.1., B.1.1.7, and B.1.351 lineages appeared, probably, quite independently. In addition, mutations common to P.1. and B.1.351 are probably associated with a rapid increase in the number of infection cases in areas where high morbidity rates were previously observed. Therefore, it is imperative to establish if there is an increased risk of re-infection in people who have had COVID-19 [87]. There is information about isolation of a new SARS-CoV-2 strain (Fin-796H) that is similar to both the British and South African variants of the virus, but identification of this variant by PCR can be difficult.

It should be noted that mutations in the *S *gene are of particular interest to researchers. The GISAID resource regularly updates data on variants of the S protein gene of the SARS-CoV-2 virus. The most common variants as of January 2021 are shown in *Fig. 1*.

An analysis of 95 full-length SARS-CoV-2 genome sequences available in GenBank for the period from December 2019 to April 2020 revealed 116 mutations, with the most frequent mutations being 8782C > T in the *ORF1ab *gene, 28144T > C in the *ORF8 *gene, and 29095C > T in the *N *gene. The identified mutations are supposed to affect the virulence and contagiousness of SARS-CoV-2 [88].

Another attempt to investigate a relationship between certain mutations in the SARS-CoV-2 genome and the virulence of the virus was made by Young *et al*. [89]. In particular, they studied how a 382-nucleotide deletion (Δ382) in the *ORF8 *region of the SARS-CoV-2 genome affects the clinical features of infection. The Δ382 variant of SARS-CoV-2 was found to be probably associated with a milder infection.

Currently, the collection and analysis of data on any relationship between mutations in the SARS-CoV-2 genome and the virulence and contagiousness of the virus is underway. The main mutations identified during the year of circulation of the pandemic virus are presented in *Fig. 1*. Obviously, a significant proportion of the mutations affecting the transmissibility of the virus are present in the gene encoding the S protein. This very important finding should be considered by developers of vaccines against SARS-CoV-2, the overwhelming majority of which are based on the S protein [90]. Sera from 20 people vaccinated with BNT162b2 (RNA vaccine encoding the S protein) was shown to neutralize SARS-CoV-2 pseudoviruses with N501 and Y501 mutations [91]. Probably, other proteins of the virus, including the nucleocapsid N protein, should be considered during the development of vaccines. For example, 90% of the epitopes in the T-cell response are located in ORF1ab of the SARS-CoV-2 nucleocapsid protein gene [92].

## SYSTEMATIZATION AND GEOGRAPHIC DISTRIBUTION OF SARS-CoV-2 GENETIC VARIANTS

Molecular genetic monitoring of the new coronavirus infection and phylogenetic analysis has enabled us to identify various genetic SARS-CoV-2 variants different in their geographic distribution. There are several approaches to a comparative genomic analysis of SARS-CoV-2 variants. One of them, proposed by Forster *et al.*, distinguishes three main SARS-CoV-2 variants (A, B, C) that differ in their amino acid substitutions. During a phylogenetic analysis, the closely related bat coronavirus BatCoVRaTG13 isolated in Yunnan Province [93] was identified as ancestral and placed at the base of the phylogenetic tree (cluster A) [94]. There are two subclusters of A which distinguish themselves by the synonymous mutation T29095C. Variant B is derived from A by two mutations: the synonymous mutation T8782C and the nonsynonymous mutation C28144T changing a leucine to a serine. In this case, type C differs from its parent type by the nonsynonymous mutation G26144T which changes a glycine into a valine [94]. Types A and C circulate mainly in Europe and America. On the contrary, type B is most prevalent in East Asia and its ancestral genome has not, apparently, spread beyond East Asia, which suggests the existence of immunological or ecological resistance to this type outside Asia [94]. These studies were complemented by the work of a group of scientists from Hong Kong [95] who performed a phylogenetic and philodynamic analysis of 247 SARS-CoV-2 genome sequences available in the GISAID database as of March 5, 2020. Among them, four genetic viral clusters, called “super-spreaders” (SSs), were identified, which were responsible for the major outbreaks of COVID-19 in various countries. For example, SS1 was widely disseminated in Asia and the United States and was mainly responsible for the outbreaks in the states of Washington and California, as well as South Korea, while SS4 contributed to the pandemic in Europe. Using the signature mutations of each SS as markers, the authors further analyzed 1,539 SARS-CoV-2 genome sequences reported after February 29, 2020 and found that 90% of these genomes were super-spreaders, with SS4 being prevalent [95]. Drawing parallels with the study [94], it should be noted that the virus identified as SS1 is equivalent to type B, SS2 is equivalent to type C, and type A is an ancestral variant. The results of a geographic distribution of different viral types are the same in both studies.

A population genetic analysis of 103 SARS-CoV-2 genomes revealed [96] that viruses may be divided into two main types (L and S) that differ in two point mutations in the amino acid sequence of site 84 (S84L) of the* ORF8 *gene. Although the L type (~70%) is more prevalent than the S type (~30%), the results of an evolutionary analysis suggest that the S type is most likely the ancestral SARS-CoV-2 version. In addition, the L type might be more aggressive and spread faster than S and human intervention may have changed the L/S ratio soon after the first outbreak of SARS-CoV-2. However, it is currently unclear whether the L type originated from the evolution of the human S type coronavirus or intermediate hosts. It is also unclear whether the L type is more virulent than the S type [96].

To assess the relationship between genetic mutations and the level of virus virulence, Zhang *et al*. analyzed clinical, molecular, and immunological data from 326 patients with a confirmed SARS-CoV-2 infection in Shanghai [97]. They identified two major clades. Clade I included several subclades characterized by differences in ORF3a: p.251G> V (subclade V) or S: p.614D> G (subclade G). Clade II differs from clade I in two linked mutations in ORF8: p.84L> S (28144T> C) and ORF1ab: p.2839S (8782C> T). This classification is inconsistent with the S/L classification [96] despite the fact that it is based on the same two related polymorphisms. In addition, the authors did not find any significant differences in the mutation rate and transmissibility in viruses belonging to clade I or II or in the clinical features of the diseases they cause.

Another approach to the systematization of genetic SARS-CoV-2 variants is offered in a preprint [98]. The authors compared viruses at a genome-wide level using the Jaccard similarity coefficient. In this case, they did not include information on the geographical origin of the samples into the analysis and did not try to model the evolutionary relationships of different SARS-CoV-2 genomes using a phylogenetic analysis. Nonetheless, the results of their analysis reflect the chronological spread of SARS-CoV-2 around the globe, from the first cases detected in China to the current outbreaks in Europe and North America. In addition, the use of the nucleotide sequences of 7,640 SARS-CoV-2 genomes presented in the GISAID database revealed that viruses cluster in four distinct genetic subgroups [98].

An analysis of tens of thousands of SARS-CoV-2 genomes, performed by a team of scientists from Temple University, identified an ancestral strain (preprint [99] published on the bioRxiv.org website). Over time, mutations in the ancestral virus genome gave rise to seven dominant lineages that spread across different continents. The use of molecular barcoding technology revealed that the genome sequences of the North American coronaviruses differed from those of the coronaviruses in circulation in Europe and Asia at that time [99].

An analysis of 75 whole genomes revealed six clusters, named Wuhan, Diamond Princess, Asian, European, USA, and Beijing [100]. Mutations in the gene encoding the spike glycoprotein S found in samples from South Korea, India, Greece, Spain, Australia, Sweden, and Yunnan may suggest a predominance of mutated strains with varying virulence.

Despite the variety of approaches to the classification of SARS-CoV-2, the GISAID consortium has developed its own generalized classification system [101] that distinguishes seven major clades (based on characteristic sets of mutations): S, L, V, G, GH, GR, and GV (*Table 2*).

**Table 2:** Modern approaches to the subspecies classification of SARS-CoV-2

Clades (GISAID [101]) and characteristic mutations		Lineages (Rambaut [103])	Clades (Hodcroft [105]) and characteristic mutations	
S	C8782T, T28144C, including NS8-L84S	A	19A and 19B	C8782T and T28144C
L	C241, C3037, A23403, C8782, G11083, G26144, T28144 (reference sequence is strain WIV04, GISAID: hCoV-19/Wuhan/WIV04/2019)	B.2	20A	C3037T, C14408T, and A23403G
V	G11083T, G26144T NSP6-L37F + NS3-G251V	B.1		
G	C241T, C3037T, A23403G, including S-D614G	B.1*		
GH	C241T, C3037T, A23403G, G25563T including S-D614G + NS3-Q57H	B.1.1.	20C	C1059T and G25563T
GR	C241T, C3037T, A23403G, G28882A, including S-D614G + N-G204R		20B	G28881A, G28882A, and G28883C
GV	C241T, C3037T, A23403G, C22227T, including S-D614G + S-A222V			

According to [68], the G and GR clades are prevalent in Europe, while S and GH are predominant in North and South America. The reference clade L is represented mainly by sequences from Asia. Currently, the clade G and its offspring, GH and GR, are the most common clades among the sequenced SARS-CoV-2 genomes, globally accounting for 74% of all known sequences. In particular, the GR clade, which carries a combination of S protein D614G and N protein RG203KR mutations, is currently the most abundant representative of SARS-CoV-2 worldwide. The original viral strain, represented by the clade L, still accounts for just 7% of the sequenced genomes [68].

An analysis of 1,566 SARS-CoV-2 genome sequences isolated in 10 Asian countries was carried out in [102]. The sequences were compared with the reference sequence of the WIV04 strain (Accession No. MN996528.1) to identify potential mutations in different regions of the genome. An *in silico *analysis showed that isolates from 10 Asian countries form clades G, GH, GR, L, S, O, and V. The highest mutation rate was detected in the GH and GR clades [102].

The GISAID classification is complemented with a more detailed, dynamic nomenclature system proposed by Rambaut *et al*. [103]. According to this system, 81 SARS-CoV-2 lineages can be distinguished, with most of them belonging to the A, B, and B.1 lineages. Six lineages derived from lineage A (A.1–A.6) and two descendant sublineages of A.1 (A.1.1 and A.3) are identified. Also, there are 16 lineages derived from lineage B. Lineage B.1, comprising 70 sublineages as of April 2020, is predominant. Lineage B.2 has six descendant sublineages. According to this classification, clades S, V, G, GH, GR, and GV correspond to lineages A, B.2, B.1, B.1*, and B.1.1 (*Table 2*) [68]. Based on this system, the pangolin software was developed [104]. It allows automatic classification of new genomes.

Another approach to systematization is described in a work by Hodcroft *et al*. [105]. The authors propose to name major clades by the year they emerged. In this case, the clade is formed from strains that have circulated for several months and have a characteristic geographic distribution. According to this classification, the following clades can currently be distinguished: 19A, 19B, 20A, 20B, and 20C (*Table 1*). Clades 19A and 19B were prevalent in Asia at the start of the pandemic, while 20A was detected in Europe in early 2020. 20B is another European clade, while 20C is a largely North American clade.

Therefore, efforts to develop a convenient and understandable classification system for the pandemic SARS-CoV-2 continue. It should be noted that at the time of preparation of our manuscript, no official ICTV guidelines for SARS-CoV-2 subspecies taxonomy had been published.

At the end of January 2020, the first cases of SARS-CoV-2 infection were detected in Russia, and since May 2020, Russia has been among the four countries with the largest number of confirmed COVID-19 cases. As of March 2021, 4.3 million cases of COVID-19 and 87,000 deaths have been reported in Russia. However, the outbreak in Russia began later than that in many neighboring European countries, possibly due to the measures taken to restrict transport links with China. A phylogenetic analysis of SARS-CoV-2 isolates from Russia showed that most samples correspond to the B.1, B.1.1, and B.1* lineages (PANGOLIN nomenclature) or to the G, GR, and GH clades (GISAID nomenclature), which are widespread in Europe [106]. In this case, the most prevalent genetic lineage is GR/20B/B.1.1 (GISAID/Nextstrain/Pangolin nomenclature, respectively) [107]. A phylogenetic analysis of Russian strains revealed that, as elsewhere, Russian SARS-CoV-2 isolates were characterized by a low mutation rate. However, a high rate of nonsynonymous mutations leading to non-conservative substitutions was found. Most of the nonsynonymous substitutions were found in nucleotide sequences encoding the N nucleoprotein. This finding may serve as indirect evidence of intensive circulation of the virus in the human population and its adaptation to new carriers [108].

## CONCLUSION

The global spread of SARS-CoV-2 with the abrupt onset of a pandemic of viral infection new to the human immune system has created conditions where it is possible to collect sufficiently convincing data on whether the structure of clinical COVID-19 forms depends on dynamic changes in the genetically determined biological properties of the virus, or if it is determined only by the characteristics of the host. This issue is fundamental to vaccine development and public health resource planning. Twelve months since the start of the spread of the new coronavirus in the human population, there is less and less doubt about the divergence of SARS-CoV-2; i.e. about the emergence of strains that differ in their biological properties, which is due to the high plasticity of the genomes of RNA viruses and favorable conditions for their evolution.

Any changes in the viral genome that disrupt the interaction with the host cell or alter the conditions of coronavirus reproduction, expression of the host’s genes, or resistance to the host’s immunity can change the degree of virus contagiousness and virulence. Furthermore, the biological properties of the virus can be altered by one or more point mutations, as has been shown in a number of studies. In this case, the interaction between the coronavirus and the host is the key to the pathogenesis of the coronavirus diseases and, ultimately, determines the outcome of the infection.
